# Food insecurity and its socioeconomic and health determinants in pregnant women and mothers of children under 2 years of age, during the COVID-19 pandemic: A systematic review and meta-analysis

**DOI:** 10.3389/fpubh.2023.1087955

**Published:** 2023-01-24

**Authors:** Francilene Maria Azevedo, Núbia de Souza de Morais, Debora Leticia Frizzi Silva, Aline Carare Candido, Dayane de Castro Morais, Silvia Eloiza Priore, Sylvia do Carmo Castro Franceschini

**Affiliations:** Department of Nutrition and Health, Federal University of Viçosa, Viçosa, Minas Gerais, Brazil

**Keywords:** pregnancy, SARS-CoV-2, postpartum period, food security, social determinants of health

## Abstract

**Background:**

The COVID-19 pandemic has reduced access to adequate food in terms of quality and quantity, especially for the most vulnerable population groups. The objective of this study was to evaluate the prevalence of Food Insecurity and its main socioeconomic and health determinants in pregnant women and mothers of children under 2 years of age, during the COVID-19 pandemic.

**Methods:**

This systematic review was conducted in accordance with the Preferred Reporting Items for Systematic Reviews and Meta-analyses (PRISMA) and registered in the International Prospective Register of Systematic Reviews (PROSPERO) (CRD42021278033). The descriptors “Pregnant Woman”, “Postpartum Women”, “Breastfeeding Women”, “COVID-19”, “Food Insecurity”, “Food Security” were combined in Scopus (Elsevier), Medline/PubMed (*via* National Library of Medicine), Embase (Elsevier), Web of Science and Science Direct independently by two researchers in September 2022. Original articles about Food Insecurity in households with pregnant women and mothers of children under 2 years of age during the COVID-19 pandemic were included. The meta-analysis of the prevalence of Food Insecurity was conducted using the RStudio software (4.0.4).

**Results:**

The initial search resulted in 539 records, and 10 articles met the proposed criteria and were included in this review. The prevalence of Food Insecurity ranged from 11.5 to 80.3% and in the meta-analysis it was 51% (IC: 30–71) (*I*^2^ = 100.0%). The main socioeconomic and health determinants were ethnicity, domain language, low education, low income, informal employment, unemployment, occurrence of mental disorders, domestic violence, in addition to the unavailability of food in markets and lack of transport. The inclusion of studies with data collection by telephone stands out as a limitation, due to the non-inclusion of vulnerable groups without access to this means of communication.

**Conclusion:**

It is necessary to implement and strengthen specific public policies for the maternal and child group with the objective of protecting and strengthening the rights of women to maintain the physical and mental integrity of this group and guarantee Food Security.

## Introduction

At the end of 2019, the first case of the disease caused by the new coronavirus (SARS-CoV-2) appeared in Wuhan, China. It is a contagious respiratory disease, later called COVID-19, which spread rapidly around the world (1, 2). In January 2020, the World Health Organization (WHO) declared the outbreak of the new coronavirus as a public health emergency of international concern, in addition to publishing guidance documents for countries regarding care in the pandemic (1, 2).

For the control of the disease, the WHO affirmed the need for rapid detection, in addition to case isolation, contact tracing, monitoring and lockdown. Thus, the most effective strategy to contain transmission is social distancing, so several countries have implemented control measures, through the temporary interruption of services considered non-essential, such as schools, religious centers, shops, public transport and events, in addition to closing borders and reducing elective health services (1, 3, 4).

Although it is the most effective measure to contain the spread of the virus, these control measures have an important socioeconomic impact, especially for the most vulnerable population (4). In low and middle-income countries, this type of restriction is not sustainable in the long term, considering that most of the population depends on face-to-face work to earn some income and have access to basic needs, such as health and food (4, 5).

Thus, the COVID-19 pandemic has worsened the food situation of individuals, especially among the neediest families. This reality is the result of the reduction or even lack of income due to the stoppage of several economic activities, which raised food prices and also contributed to families reducing the quality and quantity of food consumed (6). This context directly contributed to the increase in food insecurity worldwide (6). That can be defined when the person they lack regular access to enough safe and nutritious food for an active and healthy life. So, can be represented by the spectrum of reduced food quality, to the absence of food for basic meals, characterizing hunger (3).

According to the report by the Food and Agriculture Organization of the United Nations (FAO), between 720 and 811 million people in the world suffered from hunger in 2020, which shows an increase of about 118 million people compared to the year 2019. Assessing moderate or severe food insecurity, it was observed that it has been increasing worldwide; with prevalence of 22.6% in 2014; 26.6% in 2019 and 30.4% in 2020. It is noteworthy that among the insecure in 2020, almost 40.0% (11.9% of the world population) were in severe food insecurity (3).

Furthermore, in women, the prevalence of moderate or severe Food Insecurity is around 10% higher than in men (3). Food Insecurity has been associated with worse maternal and child health outcomes, and nutritional deficiencies in mothers and children, resulting in negative pregnancy outcomes, such as prematurity and low birth weight, as well as delay in child development that affects adulthood (7). In this regard, Chmielewska et al. highlighted that maternal group need to be protected against the prolonged effects of the pandemic. Thus, the objective of this review is to evaluate the prevalence of Food Insecurity and its socioeconomic and health determinants in pregnant women and mothers of children under 2 years of age, during the COVID-19 pandemic.

## Methods

### Study design

This systematic review and meta-analysis were performed following the recommendations of the Preferred Reporting Items for Systematic Reviews and Meta-analyses (PRISMA) (8, 9). The work was previously registered in the International Prospective Register of Systematic Reviews (PROSPERO) (CRD42021278033).

### Search strategy

This study aims to answer the question: “What is the prevalence of Food Insecurity and the socioeconomic and health determinants in pregnant women and mothers of children under 2 years of age, during the COVID-19 pandemic?”. The question was prepared according to the PECOS anagram, in which the Population (P) considered was pregnant women and mothers of children under 2 years of age, Exposure (E) was the pandemic context caused by the spread of SARS-CoV-2, without Comparison (C), the Outcome (O) considered was Food Insecurity, and the included studies (S) were observational cross-sectional and cohort studies.

To elaborate the search terms, the descriptors and synonyms were determined in the Medical Subject Headings (MeSH). Thus, the combination of the descriptors “Pregnant Woman”, “Postpartum Women”, “Breastfeeding Women”, “COVID-19”, “Food Insecurity”, “Food Security” was used with the Boolean operator AND between the terms and the OR between the synonyms of each term (Supplementary Table 1). The search was performed on September 22, 2022 by two researchers (FMA and NSM), in English, independently, without restriction of the publication period. The following bases were used Scopus (Elsevier), Medline/PubMed (*via* National Library of Medicine), Embase (Elsevier), Web of Science and Science Direct. It is noteworthy that in the Science Direct database, the filter “Research articles” was applied, which characterize the type of publication to be included. In addition, the authors performed a reverse search of the reference list of each included study to identify relevant studies that were not listed in the systematic search.

### Study selection criteria and data extraction

The selection of articles was performed independently by two authors (FMA and NSM) and disagreements were resolved by consensus. The results of searches in the databases were inserted into the software Rayyan for selection, and duplicates were identified and excluded. The initial selection took place after reading the titles and abstracts, and later the full texts. The review included original articles that evaluated Food Insecurity in pregnant women or mothers of children under 2 years of age during the COVID-19 pandemic. Publications that evaluated other physiological or age groups, reviews, study protocols, editorials and works carried out prior to the COVID-19 pandemic were not included.

The outcome evaluated was Food Insecurity, measured using psychometric scales that directly assess the food security dimension in a population, based on the perception and experience of hunger (10). Studies that used isolated questions to detect the outcome were also included. In addition, the determinants of Food Insecurity included in the inserted studies (socioeconomic and health factors) were evaluated. Furthermore, the following information was extracted from the studies: author, year of publication, geographic region, study design, sample size evaluated, objectives and main results. All data were entered into a Microsoft Office Excel data sheet.

### Quality assessment of individual studies

The quality assessment of individual studies was assessed by two authors (FMA and NSM), independently, according to the checklist of critical assessment for cross-sectional and cohort studies, recommended by The Joanna Briggs Institute (JBI) (11).

The checklist used in the evaluation of cross-sectional studies consists of eight questions, which assess the presence of inclusion criteria, sample description, adequate exposure measure, use of objective and standardized criteria to measure the outcome, identification of confounding factors, use of measures to control confounding factors, adequate measurement of results and use of appropriate statistics (11).

The checklist for longitudinal studies is composed of 11 items that assess the homogeneity of the groups included in the cohort, whether the measurement of exposure and results was validly performed, the identification of confounding factors, and strategies to deal with these factors. It also considers the absence of the outcome at the beginning of the study, if the results were validly and reliably measured, if the follow-up time was sufficient for the results to occur, if there were losses and if appropriate strategies were applied to deal with them (11).

The risk of bias for each study was classified according to the percentage of affirmative responses (“yes”), with: ≥70% considered low risk of bias, between 50 and 69% moderate and ≤49% high (12). The results of this evaluation were not used as criteria for inclusion or exclusion of studies in the systematic review.

### Statistical analysis

The prevalences of Food Insecurity presented in the studies were systematized in the meta-analysis. The generic inverse variance clustering method was used to combine prevalences from different studies into a pooled estimate. The prevalences were systematized and the meta-analysis was conducted in the software RStudio (IDE) version 4.0.4 with the metaprop function included in the meta package. In addition, the information was added in a graph with the function forest (13).

The heterogeneity between the studies was evaluated by Cochrane's Q (χ^2^ *p* < 0.10) and quantified by the *I*^2^ statistic, in which values up to 25, 50, and 75% are considered as low, moderate and high heterogeneity, respectively. The established level of statistical significance is *p* > 0.05.

## Results

**Figure 1:**
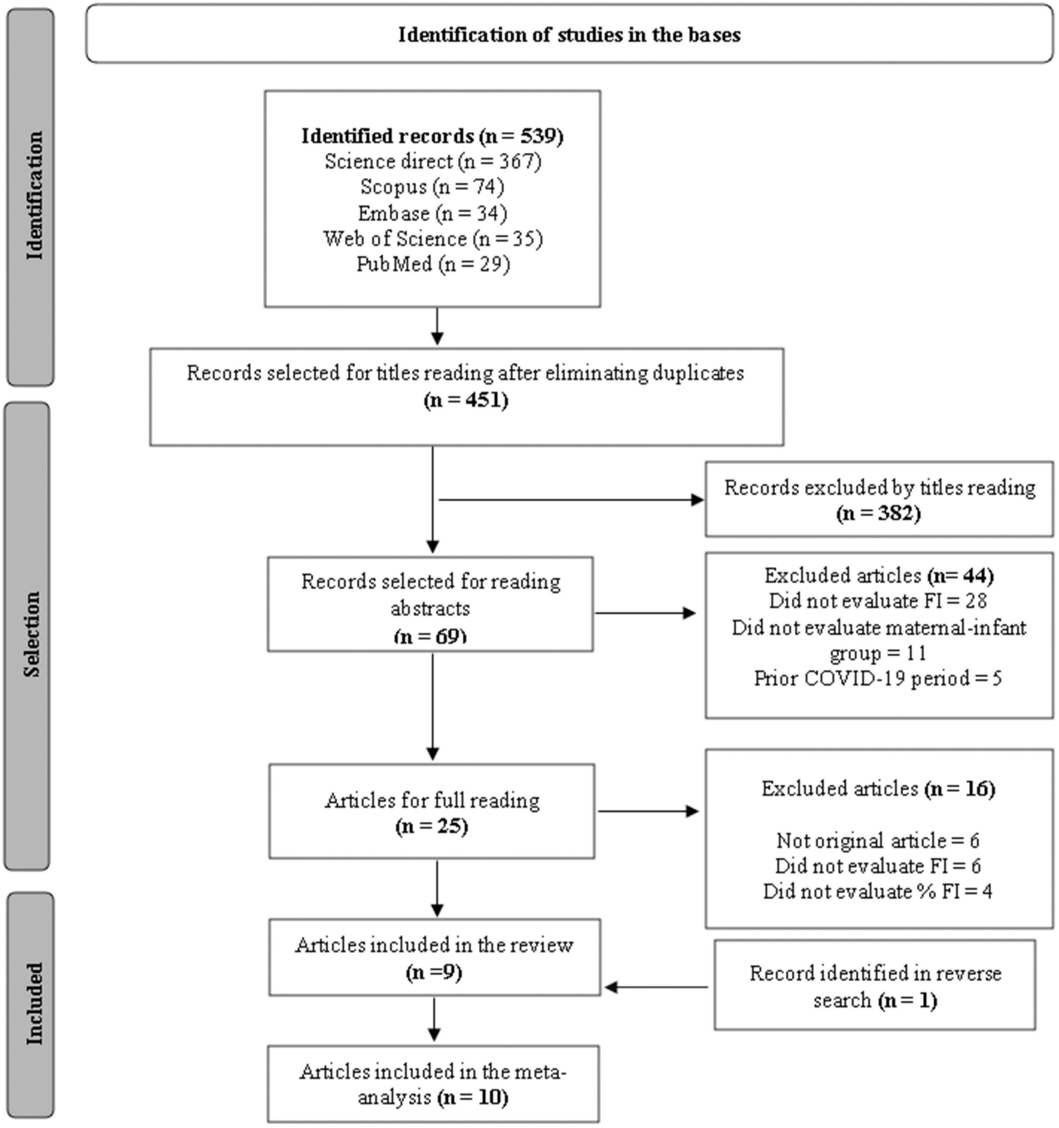
Flowchart for selecting articles according to the PRISMA methodology. FI, Food Insecurity.

The initial search resulted in 539 records. After excluding duplicates, 451 studies underwent review of titles, abstracts and full text. In the end, 10 original articles that met the inclusion criteria of this review were included, one of which was included by the reverse search (Figure 1).

The publications date from 2020 to 2022, with six cross-sectional articles (14–19) and four longitudinal articles (20–23). The studies were developed in different continents: America (14, 16–19, 23); Africa (14); Asia (15, 21, 22).

Considering the sample, the studies evaluated pregnant women (15, 17, 19), pregnant and postpartum women (14), and mothers of children under 2 years of age (16, 18, 20–23). The sample number ranged from 68 (18) to 6,592 women (17) (Table 1).

**Table 1:** Description of included studies and sample characteristics.

**Reference**	**Place**	**Type of study**	**Sample**	**Main characteristics of the sample**	**Main goal**
Abrams et al. (20)	United States	Cross-sectional	200 mothers of children < 2 years	Ethnicity: Hispanic (82.0%); Main language: Spanish (69.0%); WIC participation: 70.5% does not participate;	Assess FI during pediatric visits to federally qualified health centers during the COVID-19 pandemic.
Hamadani et al. (21)	Bangladesh	Longitudinal	2,424 mothers of children < 2 years	Education: 1–8 years of study (51.3%); Maternal occupation: Unemployment (97.3%); The prevalence of poverty (per capita < US$1·90/day) increased from 0.2 to 47.3%.	Determine the immediate impact of COVID-19 lockdown orders for women and their families in rural Bangladesh.
Abrahams et al. (14)	South Africa	Longitudinal	885 pregnant and postpartum women	Maternal occupation: Unemployment (53.6%); CMD: present in 12.4% of the sample; Maternal age: 25-35 years (54.3%).	To explore the relationship between common mental disorders, FI and experiences of domestic violence among pregnant and postpartum women attending public sector obstetric units in Cape Town during the COVID-19 pandemic.
Agampodi et al. (15)	Sri Lanka	Cross-sectional	269 pregnant women	Maternal age: 19–35 years (93.6%); Education: ≥6 years of study (99.2%); Ethnicity: Sinhalese (92.0%).	Validate the Latin American and Caribbean Scale (ELCSA) for pregnant women in Sri Lanka.
Escobar et al. (16)	United States	Cross-sectional	200 mothers of children < 2 years	Evaluated samples from 3 cohorts, and in this study, we only included data from the study that evaluated children under 2 years of age (Telomeres at Birth): Main language: English (56.8%); WIC participation: 41.4% participate.	To assess FI during COVID-19 using three prospective longitudinal studies of Latino families, compared to the time before the pandemic.
Nguyen et al. (22)	India	Longitudinal	569 mothers of children < 2 years	Maternal age: mean 25.5 (±3.8); Education: ≥ 6 years of study (86.1%); Maternal occupation: Unemployment (91.7%).	Assess changes in home FI during the pandemic and examine the interlinkages between FI with infant feeding practices and coping strategies.
Avalos et al. (17)	United States	Cross-sectional	6,592 pregnant women	Maternal age: mean 31,0 (±5.0); Ethnicity: White (37.0%); Health Insurance: Private (90.0%).	Evaluated whether COVID-19 pandemic-related health, healthcare and economic factors during pregnancy are associated with prenatal depression and anxiety.
Rosenberg et al. (18)	United States	Cross-sectional	68 mothers of neonates < 28 days	Ethnicity: Latino (52.9%); Education: ≥ 12 years of study (82.3%); Married or living with a partner (52.9%); Health Insurance: Public (63.6%).	Describe demographic characteristics and health-related social needs of families who accessed maternal infant care through a mobile medical clinic (MMC) during the COVID-19 pandemic.
Pradeilles et al. (23)	Peru	Longitudinal	244 mothers of children < 2 years	Maternal age: mean 30,0 (±6.1); Maternal occupation: Unemployment (78.3%); Government food assistance: 59.5%	Assess the impact of the pandemic on dietary outcomes of mothers and their infants and young children (IYC) in low-income urban areas of Peru.
Ridberg et al. (19)	United States	Cross-sectional	770 pregnant women	Maternal age: 26–35 years (56.0%); Ethnicity: Latina (55.0%).	Explore the impact of an ongoing supplement for fruits and vegetables provided to pregnant people enrolled in the WIC.

CMD, Common Mental Disorders; FI, Food Insecurity; WIC, Special Supplementation Nutrition Program for Women, Infants and Children.

The instruments used to assess Food Insecurity were direct methods, based on perception scales. Five studies used shortened versions: Abrams et al. (20) adopted two questions that addressed the concern that food might be lacking and the lack of money to buy food in the last 12 months; Avalos et al. (17), adopted the shortened version (2-item) of the Hunger Vital Sign screener; Rosenberg et al. (18) used the shortened version (2-item) of Household Food Security Survey (HFSS); Ridberg et al. (19), adopted a 6-item version of food security survey developed by United States Department of Agriculture; Agampodi et al. (15) developed a validation study of a reduced scale of the Latin American and Caribbean Scale (ELCSA), which is composed of eight questions (Table 2).

**Table 2:** Description of the methods used and main results related to Food Insecurity.

**Reference**	**Collection date**	**Form of collection**	**Tool used to identify FI**	**Prevalence of FI before the COVID-19 pandemic**	**Prevalence of FI during the COVID-19 pandemic**	**Main factors related to FI**
Abrams et al. (20)	April–May 2020	Interviews conducted during pediatric visits.	Screening method to identify families in FI, composed of two questions, previously validated	NR	47.0%	Higher prevalence of FI for Hispanics, Spanish-speaking and group participating in WIC.
Hamadani et al. (21)	May–June 2020	Questionnaires applied by telephone, during the pandemic period.	Household Food Insecurity Access Scale (HFIAS)	19.3%	69.4% LFI: 17.6% MFI: 36.5% SFI: 15.3%	Higher prevalence of FI in families with a father in unskilled work.
Abrahams et al. (14)	June–July 2020	Questionnaires applied by telephone, during the pandemic period.	Household Food Insecurity and Access Scale (HFIAS)	NR	80.3% LFI: 9.9% MFI: 27.4% SFI: 43.0%	There was an association between FI and CMD;
Agampodi et al. (15)	February 2020	Self-administered FI questionnaire in face-to-face collection.	Latin American and Caribbean Scale for pregnant women in Sri Lanka (ELCSA-P-SL), a shortened version of eight questions	NR	11.5%	The validated scale is feasible to efficiently screen home FI in pregnant women in Sri Lanka. The most frequent affirmative answer was the lack of food diversity. Psychological stress is associated with higher FI.
Escobar et al. (16)	May–September 2020	Questionnaires applied by telephone, during the pandemic period.	Household Food Security Scale Module, with 18 items.	NR	40,0% SAM: 13.5% LFS: 20.5% VLFS: 6.0%	Being unemployed and having less schooling were associated with higher FI. Lower prevalence of FI among families infected by COVID-19.
Nguyen et al. (22)	August 2020	Questionnaires applied by telephone, during the pandemic period.	Household Food Insecurity and Access Scale (HFIAS)	21.0%	80.0% LFI: 20.0% MFI: 30.0% SFI: 30.0%	Children in families with FI had less dietary diversity. The main factors that interfered with family food security were lack of income and unemployment, unavailability in markets and lack of transport.
Avalos et al. (17)	June–September 2020	Online self-administered questionnaire.	The validated 2-item Hunger Vital Sign screener	NR	19.0%	Individuals with food insecurity were more likely to have higher depression severity.
Rosenberg et al. (18)	April 2020	Questionnaires applied by telephone.	The validated 2-item of Household Food Security Survey (HFSS)	NR	52.9%	Health-related social needs were identified, including food insecurity, and significant anxiety related to COVID-19 transmission.
Pradeilles et al. (23)	December 2020	Questionnaires applied by telephone, during the pandemic period.	Food Insecurity Experience Scale (FIES)	NR	51.0% LFI and MFI: 46.9% SFI: 4.1,0%	Exclusive breastfeeding prevalence was higher during the COVID-19 pandemic compared to pre-COVID-19, despite remaining suboptimal (39.0%).
Ridberg et al. (19)	September 2020–June 2021	Online self-administered questionnaire.	United States Department of Agriculture (USDA) food security survey module (6-item)	NR	62.0%	This study was an intervention, however, for this review, baseline food insecurity was used. There was a reduction in food insecurity in both the group after receiving assistance.

CMD, Common Mental Disorders; FI, Food Insecurity; SFI, Severe Food Insecurity; LFI, Light Food Insecurity; MFI, Moderate Food Insecurity; LFS, Low Food Security; NR, Not Rated; MFS, Marginal Food Security; VLFS, Very Low Food Security; WIC, Special Supplementation Nutrition Program for Women, Infants and Children.

The other studies used complete scales, which allow the classification of the degree of Food Insecurity. Pradeilles et al. (23) used the full-scale version of Food Insecurity Experience Scale (FIES). The Household Food Insecurity Access Scale (HFIAS), used in three studies, is composed of nine items that assess from uncertainty regarding food availability to the experience of hunger at home in the last 30 days (14, 21, 22). The tool Household Food Security Scale Module, used by Escobar and collaborators (16), was previously validated for Latin pregnant and postpartum women residing in the United States, this is the single scale with questions covering dietary changes for children (Table 2).

The prevalence of Food Insecurity ranged from 11.5% (15) to 80.3% (14). Two articles presented the prevalence before the COVID-19 pandemic, and in both there was an expressive increase in the number of households in a situation of Food Insecurity (21, 22). Hamadami et al. (21) observed an increase from 19.3% in Food Insecurity in 2017 to 69.4% in 2020, while Nguyen and collaborators (22) reported greater changes, from 21.0% of insecure households in 2019 to 80.0% in 2020 (Table 2).

**Figure 2:**
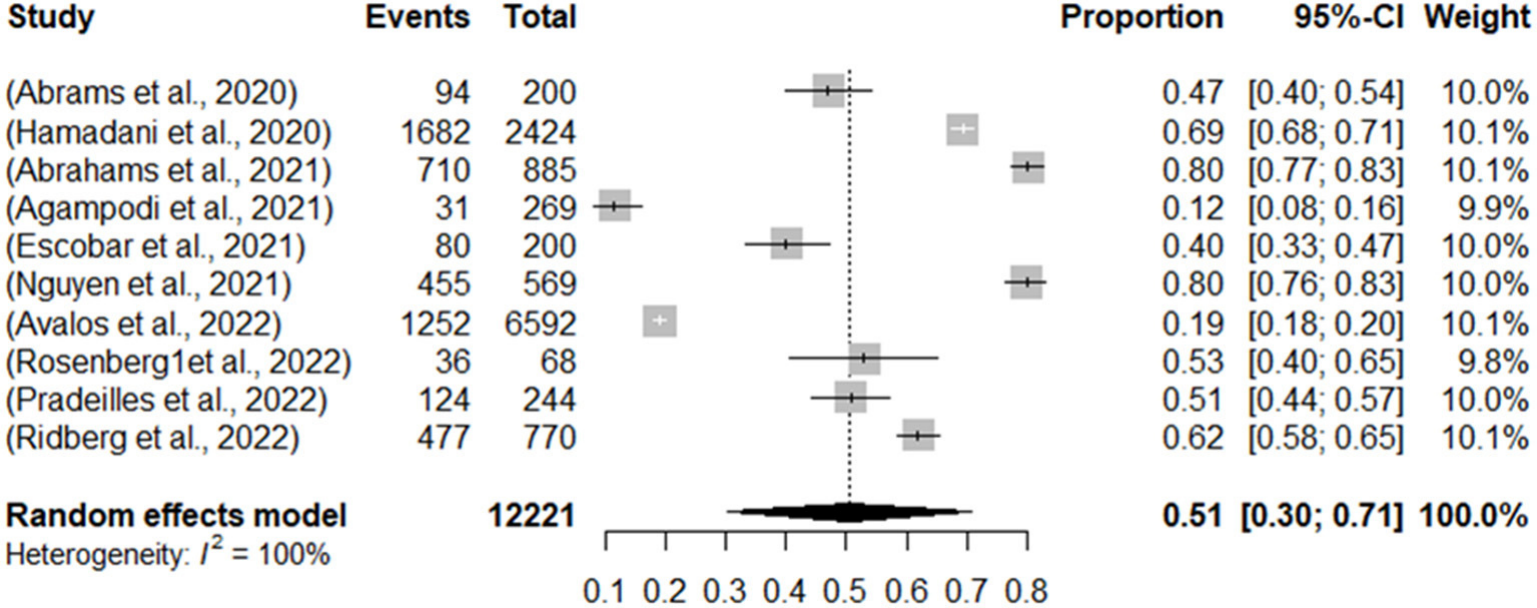
Meta-analysis of the prevalence of Food Insecurity presented in studies for pregnant women and mothers of children under 2 years of age.

The meta-analysis considered the 10 studies of the systematic review, with a total sample of 12,221 women and resulted in an overall prevalence of Food Insecurity of 51.0% (CI: 30.0–71.0%), considering the randomized effect of the samples. In general, the studies showed high heterogeneity (*I*^2^ = 100.0%), which is justified by the variation in their sample size and also by the different prevalence. In addition, different scales were used, validated for each population evaluated (Figure 2).

The main factors that were related to Food Insecurity in the studies were ethnicity (20), domain language (20), unskilled work and low maternal schooling (21, 22), unemployment and low income (22), mental disorders (14, 15, 17, 18), domestic violence (14, 15), in addition to the unavailability of food in markets and lack of transport (22) (Table 2).

**Figure 3:**
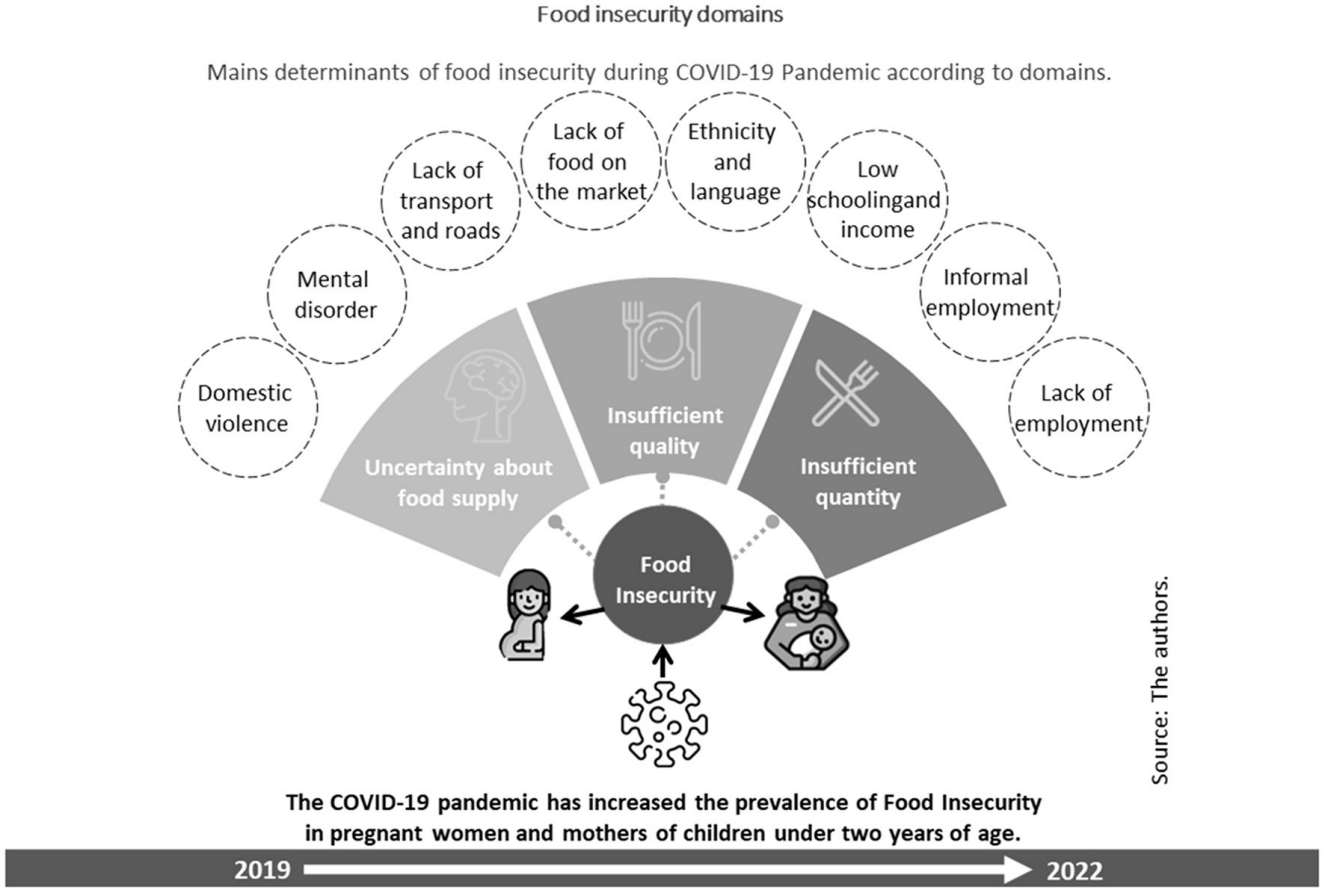
Main factors associated with Food Insecurity presented in studies for pregnant women and mothers of children under 2 years of age.

Figure 3 summarizes the points mentioned above, interrelating with the areas of Food Insecurity, including the uncertainty of buying food at home, deprivation of quality and quantity. In addition, it demonstrates that the greater the deprivation of food at home, the greater the damage to maternal and child health, which leads to greater health expenditures. It also indicates that the situation of Food Insecurity was aggravated during the period of the pandemic. The icons used in the construction of the figure are in the public domain (24).

**Figure 4:**
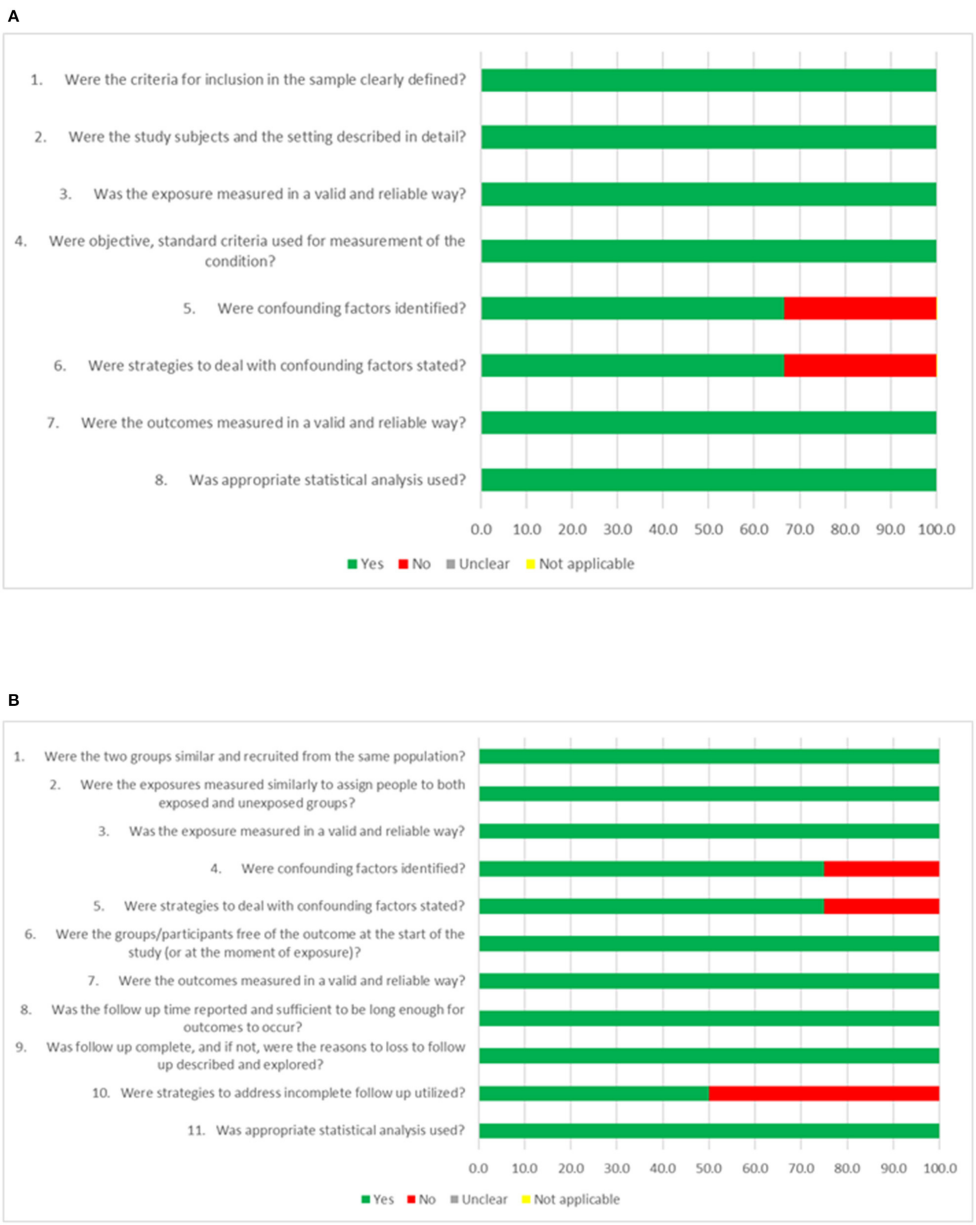
Analysis of risk of bias according to the Joanna Briggs Institute (JBI) critical assessment tools for cohort **(A)**, and cross-sectional **(B)** studies.

In the analysis of risk of bias, all studies showed low risk (percentage of affirmative answers ≥70%). For cross-sectional studies, only two did not present possible confounding factors and strategies to deal with these factors. Among the four longitudinal studies, two did not present strategies to deal with follow-up losses, and one did not identify confounding factors and strategies to deal with confounding factors. The other items of both checklists were attended (Figure 4).

## Discussion

This study presents a systematization of articles published after the beginning of the COVID-19 pandemic and shows high prevalence of Food Insecurity in the homes of pregnant women and mothers of children under 2 years old, portrayed by the result of the meta-analysis, with an estimated prevalence of 51, 0% (IC: 30.0–71.0%). Despite the high heterogeneity of the studies, we can consider this result important in relation to the global panorama of Food Insecurity in the pandemic period. In addition, the differences observed in the prevalence in each article are the result of the different contexts of social vulnerability in which the studies were carried out.

It is noteworthy that, although the articles included used tools to assess Food Insecurity, they allow making inferences about the nutritional dimension of insecurity, since they address issues related to the quality of food and its repercussions on the health of the study group.

This scenario was already expected, since the report of the United Nations Food and Agriculture Organization—FAO (3) showed that the prevalence of moderate or severe food insecurity was 10% higher among women than men in 2020, compared to the 6% difference in 2019, before the COVID-19 pandemic. Food and Nutrition Insecurity is a concern for the maternal and child group, particularly due to the damage caused by inadequate nutrition, which includes inadequate gestational weight gain that leads to low birth weight, prematurity, maternal and infant anemia, in addition to other nutritional deficiencies. After childbirth, these women are less likely to maintain Exclusive Breastfeeding (EBF), which contributes to the increase in short height, low weight for age, childhood obesity, in addition to problems that reverberate into adulthood (3).

Studies have shown that the first 1,000 days, known as the “window of opportunity”, which comprises the period from conception to 24 months after birth, are fundamental in determining health in childhood, teenagerhood and adulthood (25, 26). In the present systematic review, high prevalence of Food Insecurity were observed in pregnant women and mothers of children under 2 years of age. Therefore, there is a need for strategies to ensure adequate nutrition and provide physical and mental development since pregnancy, aimed at this period of “window of opportunity”, aiming at reducing public spending on health and education (25–27).

The health indicators mentioned above comprise global nutrition goals for 2025 and 2030, and for 2025 the objective is to: reduce the global prevalence of low birth weight from 14.6 to 10.5%; increase the prevalence of EB from 44.0 to 50.0%; reduce short stature, low weight for age and childhood obesity from 22.0, 6.7, and 5.7% to 15.4, 5.0, and 5.6%, respectively (3). These goals have become a major challenge, especially after the start of the COVID-19 pandemic, which led to the closing or reduction of health services aimed at maternal and child health, which limited access to preventive, reproductive, obstetric and child healthcare (28). In addition, there was an increase in unemployment, which culminated in a reduction in family income and less access to adequate food, balanced in quantity and quality, and available to all household members (29).

Osendarp et al. (29) demonstrated with a theoretical model the damage that the COVID-19 pandemic will cause to low and middle-income countries, with a significant increase in malnutrition and maternal and child morbidity and mortality. Food insecurity associated with reduced assistance services could result in an additional 9.3 million underweight children and 2.6 million stunted growth children, in addition to 168,000 child deaths, 2.1 million cases of maternal and long-term anemia can lead to $29.7 billion in lost productivity due to delayed child development and increased mortality at this stage (29).

The main proxy of Food Insecurity is the prevalence of individuals in poverty, which was exacerbated during the COVID-19 pandemic. Thus, the gross national income per capita of a country is not always related to the prevalence of insecurity, as it does not express the concentration of income and the consequent inequality existing in the population (30). The meta-analysis showed that among the 32 countries evaluated, those with greater inequality among their population had greater difficulties in responding to the economic impact caused by the COVID-19 pandemic (31).

Considering the latest data made available by the World Bank, it appears that among the countries evaluated in this review, the prevalence of poverty in the population (household income per capita <US$1.90/day) was higher in the India (22.0%), South Africa (18.0%), Bangladesh (14.3%), and Peru (5.8%). For the United States and Sri Lanka, the prevalence was lower, 0.9 and 1.0%, respectively (6). Furthermore, only the United States are classified as a high-income country, while South Africa and Peru comprises the middle-income category and the others are low-income (India, Bangladesh and Sri Lanka) (6). In general, the prevalence of Food Insecurity was higher in countries with a greater prevalence of poverty, with the exception of studies conducted in the United States, which evaluated ethnic minorities that are more exposed to Food Insecurity (16, 18–20). It is emphasized that 50% of the studies were carried out in the United States, and the majority of these showed high prevalence of food insecurity, showing that inequality is a factor that influences this scenario.

Another factor related to Food Insecurity that Nochaiwong et al. (31) investigated is the mental health of populations, and reported that during the pandemic there was an increase in the prevalence of various disorders. For the maternal and child public, the prevalence of mental disorders is even higher, since this moment is characterized by many doubts related to pregnancy and care for the child after birth, in addition to the insecurities caused by the pandemic context (32). In this sense, the social isolation resulting from the COVID-19 pandemic exacerbates this scenario, as the increase in food prices, associated with unemployment, generates greater instability related to the purchase of food, increasing the prevalence of Food and Nutrition Insecurity.

In this perspective, we can highlight the relationship between mental disorders and Food Insecurity (14, 15, 21). There was an increase in the prevalence of symptoms of anxiety and depression among women followed in a cohort in Bangladesh, and this parameter was related to Household Food Insecurity (21). Likewise, Abrahams et al. (14) and Agampodi et al. (15) reported a relationship between maternal psychological stress and Food Insecurity.

Another point evaluated by Hamadani et al. (21) and Abrahams et al. (14) was domestic violence suffered by women, and they observed an increase in prevalence, in addition to the relationship with Food Insecurity. Social isolation, unemployment and the implementation of the modality of home work increased the time that partners stay at home, which concomitantly contributed to an increase in domestic violence and a reduction in family income (33, 34). The two points highlighted are factors that cause insecurity and concern in women regarding the food situation at home and the ability to purchase food due to lack of stability.

Likewise, the closing of roads, the interruption of public transport and the consequent unavailability of food for sale were factors that contributed to the uncertainty about the supply of food in the households. These points also contribute to the lack of quality and diversity in the diet of families, due to interruptions in the supply of fresh and healthy food. The unavailability of food in the market and the lack of transport contribute to the reduction of food diversity and Food Insecurity (22).

The context of the pandemic highlights the ineffectiveness of food distribution systems characterized by longer supply chains and still fragmented storage, transport and services. On the other hand, short food distribution chains are resilient as they depend mainly on family labor and do not require long transport and storage, in addition to being more flexible and producing greater diversity of food (35).

Dietary diversity was also affected by the lack of income to purchase food, contributing to a worsening in the quality of diets and an increase in the consumption of low-cost sources of calories, including foods composed of simple carbohydrates and ultra-processed foods (3). Ultra-processed foods generally have a lower price, due to longer shelf life and storage, thus, the acquisition of these foods is more attractive in situations of isolation and reduced income. The studies in this review reported factors that indirectly contribute to lower family income and, consequently, to greater household Food Insecurity, which are ethnicity and maternal or head of household education (16, 20). In addition, informal jobs and unemployment were reported as determinants of Food Insecurity, both increasing in the pandemic period (16, 21, 22).

A systematic review conducted by Yashadhana et al. (36) showed that racial discrimination increases during pandemics and disadvantaged individuals are more likely to face unemployment, mainly due to language barriers. These results are in line with what was observed in this systematic review, in which even in high-income countries, the impacts arising from pandemics strongly affect ethnic minorities, which is reflected in reduced income and access to food in quantity and quality with a consequent increase in Food and Nutritional Insecurity.

Likewise, individuals with low education are predisposed to informal or lower-paid jobs and unemployment, and the conditions resulting from the COVID-19 pandemic only evidenced and aggravated this context (37). In their review, Estrela et al. (37) corroborated the provisions of the study developed by Escobar et al. (16) in which individuals exposed to contamination by the new coronavirus due to working conditions had a lower prevalence of Food Insecurity, which results from the presence of income and access to food. On the other hand, Goldman et al. (38) showed that the most socioeconomically vulnerable groups and therefore with a higher prevalence of Food Insecurity are exposed to work positions with less protection against COVID-19. Thus, this relationship must be treated with caution, since multiple factors interfere with the occurrence of Food and Nutritional Insecurity.

It is emphasized that the COVID-19 pandemic exacerbates a scenario where the maternal and child group remains vulnerable, since before the pandemic, high prevalence of food insecurity was already observed in this group. Furthermore the determinants is similar with was observed during pandemic and included social, economic and health risk factors, food access and utilization factors (39).

As limitations, the studies included in this review used different food insecurity perception scales, or isolated questions, and some do not allow the classification of degrees of insecurity. However, the use of different scales is justified by the approach of different populations. Still, the assessment of Food Insecurity in general, without classifying its degrees, is valid, since the affirmative answer to a single question on the scales already identifies the existence of Food Insecurity (40). Another limiting point is that the questionnaires were mostly administered by telephone, as a response to the challenges in conducting research during the pandemic, and this strategy may result in the non-inclusion of the most vulnerable population, especially low-income people and residents of periphery or remote rural areas without access to telephone lines.

On the other hand, the novelty of this study is highlighted by systematizing the prevalence of Food Insecurity in the maternal and child group, with a meta-analysis that allows the compilation of data from several studies and increases the statistical power of the primary research, in addition to pointing out the factors determinants of this situation during the COVID-19 pandemic and, therefore, provides useful evidence for future decision-making.

## Conclusions

This study identified high prevalence of Food Insecurity in households with pregnant women and mothers of children under 2 years of age, mainly related to the reduction of household income and unemployment, which are closely linked to the female gender, ethnic minorities and low education, contexts exacerbated during the pandemic. of COVID-19. In addition, it brings evidence related to mental disorders and domestic violence that impose a situation of general insecurity on women, which includes Food Insecurity. Also, the blockades imposed by social isolation, such as the closing of roads, led to a reduction in the variety and amount of food available in stores, in addition to raising their prices.

Specific public policies aimed at the maternal and child population are needed to reduce the high prevalence of Food Insecurity and the consequences of this situation in this period and in later stages of life. During pandemics that impose social isolation, protecting, rebuilding and strengthening women's rights through assistance services are crucial measures to maintain the physical and mental integrity of this group and guarantee Food and Nutrition Security. Considering that the pandemic has only exacerbated pre-existing inequalities, it is necessary to formulate long-term policies that guarantee access to better conditions of health, education, housing and income, especially for the most vulnerable populations, ethnic minorities and women.

## Author contributions

FA and NM: study conception and design, literature retrieval, and drafting of the manuscript. FA: data analysis and interpretation. DS, AC, and DM: critical revision and final approval of the manuscript. SF and SP: study conception, critical revision, and final approval of the manuscript. All authors contributed to the article and approved the submitted version.
