# Supplementary material for: Food insecurity and its socioeconomic and health determinants in pregnant women and mothers of children under 2 years of age, during the COVID-19 pandemic: A systematic review and meta-analysis
Source: Front Public Health. 2023 Jan 24;11:1087955. doi: 10.3389/fpubh.2023.1087955 (PMC9902714; doi:10.3389/fpubh.2023.1087955)
Supplement: Supplementary file 1 [file Table_1.DOCX]

**Supplementary Table 1. Search Terms of systematic review.**

| **Base** | | **Chave de busca** |
| --- | --- | --- |
| Web of Science  (35 results) | P | TS=(breastfeeding period) OR TS=(lactating woman) OR TS=(breastfeeding women) OR TS=(breastfeeding) OR TS=(lactating) OR TS= (pregnancy) OR TS=(pregnancies) OR TS=(gestation) OR TS=(Period, Postpartum) OR TS=(Postpartum) OR TS=(Postpartum Women) OR TS=(Women, Postpartum) OR TS=(Puerperium) OR TS=(Pregnant Woman)OR TS=(Woman, Pregnant) OR TS=(Women, Pregnant) |
|  | E | TS: (COVID 19) OR TS: (COVID-19 Virus Disease) OR TS: (COVID 19 Virus Disease) OR TS: (COVID-19 Virus Diseases) OR TS: (Disease, COVID-19 Virus) OR TS: (Virus Disease, COVID-19) OR TS: (COVID-19 Virus Infection) OR TS: (COVID 19 Virus Infection) OR TS: (COVID-19 Virus Infections) OR TS: (Infection, COVID-19 Virus) OR TS: (Virus Infection, COVID-19) OR TS: (2019-nCoV Infection) OR TS: (2019 nCoV Infection) OR TS: (2019-nCoV Infections) OR TS: (Infection, 2019-nCoV) OR TS: (Coronavirus Disease-19) OR TS: (Coronavirus Disease 19) OR TS: (2019 Novel Coronavirus Disease) OR TS: (2019 Novel Coronavirus Infection) OR TS: (2019-nCoV Disease) OR TS: (2019 nCoV Disease) OR TS: (2019-nCoV Diseases) OR TS: (Disease, 2019-nCoV) OR TS: (COVID19) OR TS: (Coronavirus Disease 2019) |
|  | O | TS: (Food Insecurity) OR TS: (Food Insecurities) OR TS: (Insecurities, Food) OR TS: (Insecurity, Food) OR TS: (food security) OR TS: (Security, Food) |
| Embase  (34 results) | P | pregnancy:ti,ab,kw OR pregnancies:ti,ab,kw OR gestation:ti,ab,kw OR 'period, postpartum':ti,ab,kw OR postpartum:ti,ab,kw OR 'postpartum women':ti,ab,kw OR 'women, postpartum':ti,ab,kw OR puerperium:ti,ab,kw OR 'pregnant woman':ti,ab,kw OR 'woman, pregnant':ti,ab,kw OR 'women, pregnant':ti,ab,kw OR 'breastfeeding period':ti,ab,kw OR 'lactating woman':ti,ab,kw OR 'breastfeeding women':ti,ab,kw OR breastfeeding:ti,ab,kw OR lactating:ti,ab,kw |
|  | E | 'covid 19':ti,ab,kw OR 'covid-19 virus disease':ti,ab,kw OR 'covid 19 virus disease':ti,ab,kw OR 'covid-19 virus diseases':ti,ab,kw OR 'disease, covid-19 virus':ti,ab,kw OR 'virus disease, covid-19':ti,ab,kw OR 'covid-19 virus infection':ti,ab,kw OR 'covid 19 virus infection':ti,ab,kw OR 'covid-19 virus infections':ti,ab,kw OR 'infection, covid-19 virus':ti,ab,kw OR 'virus infection, covid-19':ti,ab,kw OR '2019-ncov infection':ti,ab,kw OR '2019 ncov infection':ti,ab,kw OR '2019-ncov infections':ti,ab,kw OR 'infection, 2019-ncov':ti,ab,kw OR 'coronavirus disease-19':ti,ab,kw OR 'coronavirus disease 19':ti,ab,kw OR '2019 novel coronavirus disease':ti,ab,kw OR '2019 novel coronavirus infection':ti,ab,kw OR '2019-ncov disease':ti,ab,kw OR '2019 ncov disease':ti,ab,kw OR '2019-ncov diseases':ti,ab,kw OR 'disease, 2019-ncov':ti,ab,kw OR covid19:ti,ab,kw OR 'coronavirus disease 2019':ti,ab,kw OR 'disease 2019, coronavirus':ti,ab,kw OR 'sars coronavirus 2 infection':ti,ab,kw OR 'sars-cov-2 infection':ti,ab,kw OR 'infection, sars-cov-2':ti,ab,kw OR 'sars cov 2 infection':ti,ab,kw OR 'sars-cov-2 infections':ti,ab,kw OR 'covid-19 pandemic':ti,ab,kw OR 'covid 19 pandemic':ti,ab,kw OR 'covid-19 pandemics':ti,ab,kw OR 'pandemic, covid-19' |
|  | O | 'food insecurity':ti,ab,kw OR 'insecurity, food':ti,ab,kw OR 'food insecurities':ti,ab,kw OR 'insecurities, food':ti,ab,kw OR 'insecurity, food':ti,ab,kw OR 'security, food':ti,ab,kw OR 'food security':ti,ab,kw |
| PubMed  (29 results) | P | ‘breastfeeding period’[Title/Abstract] OR ‘lactating woman’[Title/Abstract] OR ‘breastfeeding women’[Title/Abstract] OR breastfeeding [Title/Abstract] OR lactating [Title/Abstract] OR Pregnancy[Title/Abstract] OR Pregnancies[Title/Abstract] OR Gestation[Title/Abstract] OR ‘Period, Postpartum’[Title/Abstract] OR Postpartum[Title/Abstract] OR ‘Postpartum Women’[Title/Abstract] OR ‘Women, Postpartum’[Title/Abstract] OR Puerperium[Title/Abstract] OR ‘Pregnant Woman’[Title/Abstract] OR ‘Woman, Pregnant’[Title/Abstract] OR ‘Women, Pregnant’[Title/Abstract] |
|  | E | (((((((((((((((((((((((((((((((((((COVID 19) OR (COVID-19 Virus Disease)) OR (COVID 19 Virus Disease)) OR (COVID-19 Virus Diseases)) OR (Disease, COVID-19 Virus)) OR (Virus Disease, COVID-19)) OR (COVID-19 Virus Infection)) OR (COVID 19 Virus Infection)) OR (COVID-19 Virus Infections)) OR (Infection, COVID-19 Virus)) OR (Virus Infection, COVID-19)) OR (2019-nCoV Infection)) OR (2019 nCoV Infection)) OR (2019-nCoV Infections)) OR (Infection, 2019-nCoV)) OR (Coronavirus Disease-19)) OR (Coronavirus Disease 19)) OR (2019 Novel Coronavirus Disease)) OR (2019 Novel Coronavirus Infection)) OR (2019-nCoV Disease)) OR (2019 nCoV Disease)) OR (2019-nCoV Diseases)) OR (Disease, 2019-nCoV)) OR (COVID19)) OR (Coronavirus Disease 2019)) OR (Disease 2019, Coronavirus)) OR (SARS Coronavirus 2 Infection)) OR (SARS-CoV-2 Infection)) OR (Infection, SARS-CoV-2)) OR (SARS CoV 2 Infection)) OR (SARS-CoV-2 Infections)) OR (COVID-19 Pandemic)) OR (COVID 19 Pandemic)) OR (COVID-19 Pandemics)) OR (Pandemic, COVID-19))) |
|  | O | ‘Food Insecurity’[Title/Abstract] OR ‘Food Insecurities’[Title/Abstract] OR ‘Insecurities, Food’[Title/Abstract] OR ‘Insecurity, Food’[Title/Abstract] OR 'security, food'[Title/Abstract] OR 'food security'[Title/Abstract] |
| Scopus  (74 results) | P | ( TITLE-ABS-KEY ( breastfeeding AND period ) OR TITLE-ABS-KEY ( lactating AND woman ) OR TITLE-ABS-KEY ( breastfeeding AND women ) OR TITLE-ABS-KEY ( breastfeeding ) OR TITLE-ABS-KEY ( lactating ) OR TITLE-ABS-KEY ( pregnancy ) OR TITLE-ABS-KEY ( pregnancies ) OR TITLE-ABS-KEY ( gestation ) OR TITLE-ABS-KEY ( period, AND postpartum ) OR TITLE-ABS-KEY ( postpartum ) OR TITLE-ABS-KEY ( postpartum AND women ) OR TITLE-ABS-KEY ( women, AND postpartum ) OR TITLE-ABS-KEY ( puerperium ) OR TITLE-ABS-KEY ( pregnant AND woman ) OR TITLE-ABS-KEY ( woman, AND pregnant ) OR TITLE-ABS-KEY ( women, AND pregnant ) ) |
|  | E | ( TITLE-ABS-KEY ( covid-19 ) OR TITLE-ABS-KEY ( covid 19 ) OR TITLE-ABS-KEY ( covid-19 AND virus AND disease ) OR TITLE-ABS-KEY ( covid 19 virus AND disease ) OR TITLE-ABS-KEY ( covid-19 AND virus AND diseases ) OR TITLE-ABS-KEY ( disease, AND covid-19 AND virus ) OR TITLE-ABS-KEY ( virus AND disease, AND covid-19 ) OR TITLE-ABS-KEY ( covid-19 AND virus AND infection ) OR TITLE-ABS-KEY ( covid 19 virus AND infection ) OR TITLE-ABS-KEY ( covid-19 AND virus AND infections ) OR TITLE-ABS-KEY ( infection, AND covid-19 AND virus ) OR TITLE-ABS-KEY ( virus AND infection, AND covid-19 ) OR TITLE-ABS-KEY ( 2019-ncov AND infection ) OR TITLE-ABS-KEY ( 2019 ncov AND infection ) OR TITLE-ABS-KEY ( 2019-ncov AND infections ) OR TITLE-ABS-KEY ( infection, AND 2019-ncov ) OR TITLE-ABS-KEY ( coronavirus AND disease-19 ) OR TITLE-ABS-KEY ( coronavirus AND disease 19 ) OR TITLE-ABS-KEY ( 2019 novel AND coronavirus AND disease ) OR TITLE-ABS-KEY ( 2019 novel AND coronavirus AND infection ) OR TITLE-ABS-KEY ( 2019-ncov AND disease ) OR TITLE-ABS-KEY ( 2019 ncov AND disease ) OR TITLE-ABS-KEY ( 2019-ncov AND diseases ) OR TITLE-ABS-KEY ( disease, AND 2019-ncov ) OR TITLE-ABS-KEY ( covid19 ) OR TITLE-ABS-KEY ( coronavirus AND disease 2019 ) OR TITLE-ABS-KEY ( disease AND 2019, AND coronavirus ) OR TITLE-ABS-KEY ( sars AND coronavirus 2 infection ) OR TITLE-ABS-KEY ( sars-cov-2 AND infection ) OR TITLE-ABS-KEY ( infection, AND sars-cov-2 ) OR TITLE-ABS-KEY ( sars AND cov 2 infection ) OR TITLE-ABS-KEY ( sars-cov-2 AND infections ) OR TITLE-ABS-KEY ( covid-19 AND pandemic ) OR TITLE-ABS-KEY ( covid 19 pandemic ) OR TITLE-ABS-KEY ( covid-19 AND pandemics ) OR TITLE-ABS-KEY ( pandemic, AND covid-19 ) ) |
|  | O | (TITLE-ABS-KEY ( food AND insecurity ) OR TITLE-ABS-KEY ( food AND insecurities ) OR TITLE-ABS-KEY ( insecurities, AND food ) OR TITLE-ABS-KEY ( insecurity, AND food ) OR TITLE-ABS-KEY ( security, AND food ) OR TITLE-ABS-KEY ( food AND security)) |
| Science direct  (367) | P  E  O | (breastfeeding OR lactating OR Pregnancy OR Postpartum) AND (COVID 19) AND ('Food Insecurity' OR 'Food Security')  **This base limits the number of search terms.** |
